# Early childhood development in Rwanda: a policy analysis of the human rights legal framework

**DOI:** 10.1186/s12914-016-0076-0

**Published:** 2016-01-12

**Authors:** Agnes Binagwaho, Kirstin W. Scott, Sardis H. Harward

**Affiliations:** Ministry of Health, Republic of Rwanda, Kigali, Rwanda; Harvard Medical School, Boston, MA USA; Geisel School of Medicine at Dartmouth College, Hanover, NH USA; The Dartmouth Institute for Health Policy and Clinical Practice, Lebanon, NH USA

**Keywords:** Early childhood development, Rwanda, Human rights

## Abstract

**Background:**

Early childhood development (ECD) is a critical period that continues to impact human health and productivity throughout the lifetime. Failing to provide policies and programs that support optimal developmental attainment when such services are financially and logistically feasible can result in negative population health, education and economic consequences that might otherwise be avoided. Rwanda, with its commitment to rights-based policy and program planning, serves as a case study for examination of the national, regional, and global human rights legal frameworks that inform ECD service delivery.

**Discussion:**

In this essay, we summarize key causes and consequences of the loss of early developmental potential and how this relates to the human rights legal framework in Rwanda. We contend that sub-optimal early developmental attainment constitutes a violation of individuals’ rights to health, education, and economic prosperity. These rights are widely recognized in global, regional and national human rights instruments, and are guaranteed by Rwanda’s constitution. Recent policy implementation by several Rwandan ministries has increased access to health and social services that promote achievement of full developmental potential. These ECD-centric activities are characterized by an integrated approach to strengthening the services provided by several public sectors. Combining population level activities with those at the local level, led by local community health workers and women’s councils, can bolster community education and ensure uptake of ECD services.

**Conclusions:**

Realization of the human rights to health, education, and economic prosperity requires and benefits from attention to the period of ECD, as early childhood has the potential to be an opportunity for expedient intervention or the first case of human rights neglect in a lifetime of rights violations. Efforts to improve ECD services and outcomes at the population level require multisector collaboration at the highest echelons of government, as well as local education and participation at the community level.

## Background

The demographic characteristics of many developing nations imply that interventions targeting the period of early childhood development (ECD) have tremendous potential to promote population health and socioeconomic development. Rwanda is an east African nation of approximately 12 million people, over 1.5 million of whom are between 0 and 3 years of age [1]. With a fertility rate of 4.6 births per woman in 2012, the population that stands to benefit from early childhood development (ECD) interventions is expected to grow over the next several years [2].

Loss of early developmental potential from any cause continues to manifest throughout an individual’s lifetime as health, social, educational and economic consequences [3, 4]. Sub-optimal ECD thus constrains individuals’ ability to exercise specific human rights enshrined in documents such as the Universal Declaration of Human Rights (UDHR), the International Covenant on Economic, Social and Cultural Rights (ICESCR) and the Convention on the Rights of the Child (CRC), among others [5–7]. In developing nations in particular, the consequences of impediments to ECD contribute to intergenerational transmission of poverty and attendant human rights violations [3, 4].

As a signatory to the ICESCR, African Charter on Human and People’s Rights (ACHPR), CRC, African Charter on the Rights and Welfare of the Child (ACRWC) and numerous other international human rights documents, Rwanda is legally bound to recognize all rights described therein. Furthermore, Rwanda’s own constitution entitles citizens to “the right to physical and mental integrity,” and “to a healthy and satisfying environment” [8]. The same document holds the government of Rwanda responsible for “mobilizing the population for activities aimed at promoting good health and assisting in the implementation of these activities” [8].

This essay examines local and global human rights legal frameworks related to ECD, argues that factors that limit early developmental attainment impinge on the human rights to health, education and economic prosperity, and discusses how Rwandan children access their right to ECD. Rwanda was selected for in depth analysis due to the authors’ extensive experience working in the country and the nation’s demonstrated will and ability to address children’s health issues. Our objective is not to provide a comprehensive review or analysis of all factors affecting early developmental gain or loss. Rather, we summarize relevant findings from the ECD literature to contextualize national and international reports and policies influencing Rwanda’s ECD legal framework. By assessing ECD rights and opportunities in the context of knowledge and policy, we hope to inform national and international discussions and motivate future research into integrated, rights-based policy approaches to fostering full developmental attainment.

## Discussion

### Early childhood development in international legal context

Although ECD is not mentioned explicitly in the UDHR, ICESCR, CRC, ACHPR or ACRWC, references to the health, educational, and economic consequences of developmental attainment and/or loss are quite frequent.

The ICESCR and the ACHPR describe “the right of everyone to the enjoyment of the highest attainable standard of physical and mental health,” a concept that is further developed in the 1978 Declaration of Alma-Ata [7, 9, 10]. This Declaration is unique in that it defines health as “not merely the absence of disease or infirmity,” but “a state of complete physical, mental and social wellbeing” [10]. The consequences of poor ECD on child and adult health are, therefore, violations of human rights to the extent that they result in any state less than the highest level of well-being attainable with optimal early development. While certainly necessary, and in some places, a feat in and of itself, merely surviving childhood is not sufficient to exercise one’s right to early development. Any negative impact on health due to factors encountered during the first 3 years of life, regardless of when it presents, represents an infringement on an individual’s right to full developmental potential manifesting as a violation of that individual’s right to health.

If not the most proximal forces affecting health status, education and economic prosperity are perhaps the most influential and potentially pernicious social determinants of health. The human rights to education and economic prosperity are expressed in the ACHPR, UDHR and ICESCR, the latter two of which declare that education in particular should “be directed to the full development of the human personality” [6, 7, 9]. The relationship between educational attainment and economic prosperity is quite intuitive; as a general rule, educational achievement predicts earning potential and earning potential predicts economic security [7, 11]. And indicators of early developmental attainment during childhood affect numerous educational outcomes, including school attendance, academic performance, and highest grade completed [4, 5, 11, 12]. Loss of developmental potential in early childhood, as a result of health complications or otherwise, thereby predisposes children to inability to access their right to education, and ultimately, to economic prosperity.

In addition to addressing the consequences of loss of early developmental potential, several international legal instruments discuss the special attention and protections warranted by the vulnerability of childhood, including explicit references to a child’s right to full development [8, 13]. Both the CRC and ACRWC bind States Parties to the responsibility to “ensure to the maximum extent possible the survival and development of the child”; the CRC extends the duration of this responsibility to include the period “before as well as after birth” [8, 13]. This additional stipulation deserves special consideration, as it gestures to the potential for intergenerational transmission of developmental attainment. In order to protect a child before birth, certain protections must be extended to that child’s caregivers and community. If caregivers and communities are afflicted with ill health, minimal education and economic insecurity, the risk of losing early developmental potential among future generations increases substantially. Loss of developmental potential does not, therefore, result from sporadic instances of misfortune, but rather from chronic human rights violations that persist for generations. In this sense, loss of early developmental potential is both a product and an origin of human rights violations. Restoring opportunities for optimal ECD thereby both realizes rights for the individuals involved, and reduces the potential for further rights violations in the future.

### Early childhood development in Rwandan legal context

In order to adequately assume these bold global health and human rights responsibilities and offer high quality medical services within the bounds of financial constraints, the Government of Rwanda (GoR) has developed a decentralized approach to program planning and implementation that emphasizes local needs assessments, problem-solving, accountability and solidarity [14]. In particular, the Ministry of Health (MoH) has adopted a service delivery structure that relies heavily upon community health workers (CHWs) and local health centers [15].

This locally driven structure, created as a result of the national decentralization law, coupled with the financially protective effects of the *Mutuelles de la Santé* insurance program and pay-for-performance compensation schemes have yielded astounding improvements in population health, particularly among young children [15, 16]. Since 2005, malaria deaths among children under five have decreased by more than 75 %, mother-to-child transmission of HIV has been reduced to less than 2 %, and immunization coverage has reached levels that many developed nations would envy [17–19].

Rwanda’s health sector is thus uniquely situated to contribute to addressing current gaps in ECD services. With CHWs on the front lines of care delivery, the health sector has the ability to actively seek pregnant women, connect them to appropriate health services and track them via a novel SMS-based system through all necessary follow-up [15, 16, 20, 21]. The health sector is also the first to interact with newborn children through delivery and subsequent preventive care services, and the first to intervene with curative and palliative care in the event of illness [15, 16]. Expanding currently offered health services to include ECD education for caregivers—particularly if undertaken in conjunction with community-based services provided by the Ministry of Education, the Ministry of Gender and Family Promotion, and other sectors—has the potential to dramatically improve ECD outcomes.

The achievements made in Rwanda’s education sector have also been remarkable: investments in public schools and teacher training have expanded access to education such that 98.7 % of primary school-aged children were enrolled in school in 2012 [2, 22]. Furthermore, the gender disparities in school enrollment that plague similarly resource-limited settings are no longer present in Rwanda: gender equity has been achieved at both primary and secondary levels of education [2]. These investments in education have been made with poverty reduction and national development in mind, goals that are explicitly articulated in Rwanda’s Vision 2020 [23].

Yet while these improvements in health outcomes and access to education indicate that Rwanda has already accomplished some measure of success in reducing exposure to risk factors for loss of early developmental potential, there is still room for continued progress. Poverty and malnutrition remain highly prevalent, with 44.9 % of the population living at or under the national poverty line in 2011 and an estimated stunting prevalence of 44.3 % in 2010 [11, 17]. Innovative policies and interventions will be necessary to reduce the burden of these risk factors for loss of early developmental potential and to truly capitalize on the benefits of optimal ECD.

Rwanda’s national ECD policy and strategic plan represent one such innovative approach to integrated and multisector ECD service delivery [24, 25]. Recently under the purview of the Ministry of Education, but now transferred to the Ministry of Gender and Family Promotion, Rwanda’s ECD policy emphasizes the role of each sector—health, education, finance, family and gender promotion, and local government—in optimizing early developmental attainment in Rwanda [24]. The policy and strategic plan both recognize the potential of existing programs such as the health sector’s CHW network and the education sector’s public school system to participate in ECD programming. Additional community-based components will be necessary to fully capture the potential of local human resources, however.

One community-based source of support for ECD activities can be found in the recent election of women’s councils at the village level, supported by the Ministries of Local Administration and Gender and Family Promotion. These volunteer councils champion women’s issues, support expectant and new mothers, raise ECD awareness, and promote interaction between parents and children. In doing so, these women have the potential to foster an environment at the village level that provides support to children and families in order to facilitate full developmental attainment [26]. Such activities, supported by multisector collaboration among the Social Cluster Ministries, realize ECD both as a human right and as an instrument to promote the rights to health, education, and economic prosperity.

### Limitations

This essay focuses exclusively on the Rwandan context and did not include a formal systematic review of the literature on ECD, thereby limiting the generalizability of the analysis as well as its rigor. Though the purpose of the essay was to examine the ECD legal framework in Rwanda, several other nations are making noteworthy strides toward strengthening their ECD programs and deserve acknowledgement for these achievements. While common principles such as multisector collaboration have the potential to strengthen ECD service delivery everywhere, optimal approaches to promoting full developmental attainment must be unique to each context.

## Conclusions

The period of early childhood development is at once one of the most crucial and most vulnerable stages of an individual’s life. Myriad influences threaten loss of early developmental potential, manifesting as immediate or delayed health, educational and economic consequences. Factors limiting development during early childhood thus constitute human rights violations insofar as they constrain individuals’ ability to exercise their internationally recognized human rights to health, education and economic prosperity at any time in life. What is worse, the propagation of these risk factors in communities condemns future generations to similar circumstances. Innovative and integrated strategies based upon proven methods of expanded care delivery—such as Rwanda’s massively successful CHW program and the involvement of locally elected women in promoting appropriate practices—must be carefully considered by policymakers developing national plans to realize ECD as a right, and to create supportive systems that allow children and communities to exercise this right. We therefore call upon Rwanda, and all nations suffering from similar losses of developmental potential, to recognize and ensure the right of all to maximal development during early childhood.

## Abbreviations

ACHPR: African Charter on Human and People’s Rights
ACRWC: African Charter on the Rights and Welfare of the Child
CRC: Convention on the Rights of the Child
ECD: Early childhood development
ICESCR: International Covenant on Economic, Social and Cultural Rights
UDHR: Universal Declaration of Human Rights
